# Understanding the Molecular Landscape of Endometriosis: A Bioinformatics Approach to Uncover Signaling Pathways and Hub Genes

**DOI:** 10.5812/ijpr-144266

**Published:** 2024-04-06

**Authors:** Junhua Tian, Xiaochun Liu

**Affiliations:** 1 Department of Gynecology, Shanxi Bethune Hospital, Shanxi Academy of Medical Sciences, Tongji Shanxi Hospital, Third Hospital of Shanxi Medical University, Taiyuan, China

**Keywords:** Endometriosis, Microarray, Hub Genes, Molecular Docking, Bioinformatics Analysis

## Abstract

**Background:**

Endometriosis is a chronic gynecological disorder characterized by the ectopic growth of endometrial tissue outside the uterus, leading to debilitating pain and infertility in affected women. Despite its prevalence and clinical significance, the molecular mechanisms underlying the progression of endometriosis remain poorly understood. This study employs bioinformatics tools and molecular docking simulations to unravel the intricate genetic and molecular networks associated with endometriosis progression.

**Objectives:**

The primary objectives of this research are to identify differentially expressed genes (DEGs) linked to endometriosis, elucidate associated biological pathways using the Database for Annotation, Visualization, and Integrated Discovery (DAVID), construct a Protein-Protein Interaction (PPI) network to identify hub genes, and perform molecular docking simulations to explore potential ligand-protein interactions associated with endometriosis.

**Methods:**

Microarray data from Homo sapiens, specifically Accession: GDS3092 Series = GSE5108 (Platform: GPL2895), were retrieved from the NCBI Gene Expression Omnibus (GEO). The data underwent rigorous preprocessing and DEG analysis using NCBI GEO2. Database for Annotation, Visualization, and Integrated Discovery analysis was employed for functional annotation, and a PPI network was constructed using the STITCH database and Cytoscape 3.8.2. Molecular docking simulations against target proteins associated with endometriosis were conducted using MVD 7.0.

**Results:**

A total of 1 911 unique elements were identified as DEGs associated with endometriosis from the microarray data. Database for Annotation, Visualization, and Integrated Discovery analysis revealed pathways and biological characteristics positively and negatively correlated with endometriosis. Hub genes, including BCL2, CCNA2, CDK7, EGF, GAS6, MAP3K7, and TAB2, were identified through PPI network analysis. Molecular docking simulations highlighted potential ligands, such as Quercetin-3-o-galactopyranoside and Kushenol E, exhibiting favorable interactions with target proteins associated with endometriosis.

**Conclusions:**

This study provides insights into the molecular signatures, pathways, and hub genes associated with endometriosis. Utilizing DAVID in this study clarifies biological pathways associated with endometriosis, revealing insights into intricate genetic networks. Molecular docking simulations identified ligands for further exploration in therapeutic interventions. The consistent efficacy of these ligands across diverse targets suggests broad-spectrum effectiveness, encouraging further exploration for potential therapeutic interventions. The study contributes to a deeper understanding of endometriosis pathogenesis, paving the way for targeted therapies and precision medicine approaches to improve patient outcomes. These findings advance our understanding of the molecular mechanisms in endometriosis (EMS), offering promising avenues for future research and therapeutic development in addressing this complex condition.

## 1. Background

Endometriosis is a complex and debilitating gynecological disorder that affects millions of women worldwide, characterized by the presence of endometrial-like tissue outside the uterine cavity, primarily within the pelvic region (1). This condition often leads to chronic pelvic pain, infertility, and a decreased quality of life. Despite its significant impact on women's health, the precise molecular mechanisms underlying the development and progression of endometriosis remain poorly understood (2). The pathogenesis of endometriosis is thought to involve multiple factors, including genetic, hormonal, and immunological components (3). Recent advancements in molecular biology and bioinformatics have provided new opportunities to explore the intricate genetic and molecular networks underlying this condition (4). High-throughput technologies, such as next-generation sequencing (NGS) and microarray analyses, have enabled the generation of vast amounts of omics data, offering unprecedented insights into the genes and pathways associated with endometriosis (5, 6). This research aims to leverage bioinformatics tools and techniques to analyze the extensive datasets available on endometriosis. By integrating genomics, transcriptomics, proteomics, and other -omics data, we seek to identify crucial genes and pathways that play pivotal roles in the development, progression, and potentially the treatment of endometriosis (7). Understanding the molecular basis of endometriosis is critical not only for shedding light on the disease's etiology but also for discovering potential biomarkers and therapeutic targets (8). In this study, we will conduct a comprehensive bioinformatics analysis, including differential gene expression analysis, pathway enrichment analysis, protein-protein interaction network analysis, and functional annotation, to elucidate the molecular signatures associated with endometriosis (9). By examining data from diverse sources and cohorts, we aim to identify commonalities and distinctions in gene expression patterns and pathway dysregulation across different stages and phenotypes of endometriosis (10). The outcomes of this research hold the potential to uncover novel insights into the molecular mechanisms driving endometriosis and offer a foundation for the development of targeted therapies and precision medicine approaches for individuals affected by this enigmatic disease (11). The novelty of this study lies in its multi-faceted exploration of endometriosis, combining gene expression analysis, pathway elucidation, hub gene identification, and molecular docking simulations. This integrative approach contributes to a more comprehensive understanding of the molecular mechanisms underlying endometriosis and provides potential directions for future research and therapeutic development. Ultimately, this work may contribute to improving the diagnosis, management, and overall quality of life for women suffering from endometriosis.

## 2. Methods

The present study on the progression of endometriosis involved bioinformatics analyses such as Data Preprocessing and Differentially Expressed Gene (DEG) analysis, Database for Annotation, Visualization, and Integrated Discovery (DAVID) analysis, and Protein-Protein Interaction (PPI) interaction analysis. The DAVID analysis begins with the submission of gene lists, which then undergo analysis through a range of text and pathway-mining tools available on the platform.

### 2.1. Data Resource

The expression microarray datasets associated with endometriosis (EMS) in Homo sapiens were retrieved from the NCBI repository Gene Expression Omnibus (GEO), which includes high-throughput microarray datasets with accession number Accession: GDS3092 Series = GSE5108 (Platform: GPL2895). These datasets comprised a total of 22 expression profiling assay samples.

### 2.2. Data Preprocessing and Differentially Expressed Gene Analysis

The retrieved microarray data for differentially expressed genes (DEGs) was analyzed using NCBI GEO2. Differentially expressed genes were determined based on a significant cutoff with a P-value < 0.001 and a log-fold change < −0.5 or > 0.5.

### 2.3. Database for Annotation, Visualization, and Integrated Discovery Analysis

The DAVID was analyzed using DAVID 6.8. The biological pathways involved in EMS were analyzed, and processes related to the DEGs were conducted using pathway enrichment analysis in DAVID 6.8. Statistical significance was set with a cutoff value of P < 0.05. The analysis in DAVID begins with the submission of a gene list capable of accommodating various common gene identifiers (12). Subsequently, this gene list undergoes analysis through a range of text and pathway-mining tools available on the platform, offering diverse functionalities, including gene functional classification, the creation of functional annotation charts, and facilitating clustering and functional annotation tables (13).

### 2.4. PPI Interaction Analysis

The PPI network of the DEGs associated with EMS was mapped using the STITCH database and Cytoscape 4.0, and the core targets of EMS were constructed using the STITCH database. The analysis may provide functional annotations of the proteins associated with the progression of EMS. The network map may also aid in targeting specific proteins or enzymes by inhibiting their function.

### 2.5. Traditional Chinese Medicine Chemical Compounds

A search was conducted on the Traditional Chinese Medicine Systems Pharmacology Database and Analysis Platform (TCMSP, http://tcmspw.com/index.php) to identify the principal chemical compounds linked to treating breast cancer. The three-dimensional geometries of these compounds were obtained from the NCBI PubChem database (https://pubchem.ncbi.nlm.nih.gov/) and subsequently optimized through the application of standard molecular force fields, such as MM2, using ChemOffice 2010 (PerkinElmer, USA).

### 2.6. Molecular Docking of Target Proteins Associated with EM

Molecular docking simulations were conducted against five protein targets associated with endometriosis, utilizing MVD 7.0 (Molexus IVS, Denmark), which employs a grid-based docking approach. This method subdivides the binding site of the target molecule into a grid of points, calculating the binding energy at each grid point. In this study, MVD is employed to predict and optimize the binding of small molecules (ligands) to target proteins. Initially, the binding cavity and active site of endometriosis-associated protein targets were predicted, and the 3D structures of the target proteins were optimized using the Protein Preparation Tool. This is because identifying the binding cavity and active site aids in identifying the potential targets of endometriosis-associated proteins. It provides critical information for designing and optimizing potential drug candidates, which is crucial for understanding the interaction between the target protein and potential drug molecules. The binding sites of target proteins were determined using a grid-based cavity prediction algorithm. Initially, a protein-covering discrete grid is generated with a resolution of 0.8 Å. At each grid point, a sphere of 1.4 Å radius is positioned, and its potential overlap with spheres determined by the Van der Waals radii of protein atoms is examined. Subsequently, each accessible grid point is assessed to determine if it contributes to a cavity, progressing until the grid boundaries are reached. The final step involves identifying connected regions, where two grid points are considered connected if they are neighbors. Regions with a volume less than 10.0 Å³ are excluded as irrelevant. However, the up-regulation of a protein does not necessarily mean it should be inhibited, as some proteins may have protective roles or be part of adaptive responses. The 3D structures of the target proteins were optimized using the Protein Preparation Tool. Bond flexibility and side chain flexibility of the protein were set to standard values (tolerance = 1.0 and strength = 0.90). The RMSD threshold was established at 2.00 Å with 1 000 iterations and 50 iterations for simplex evolution size. Notably, MVD accommodates both flexible ligands and flexible protein receptors, providing a more accurate representation of the real-world binding process, where conformational changes in both the ligand and receptor may occur upon binding.

## 3. Results

### 3.1. Identification of Microarray Data

In the present study, a total of 277 404 microarray elements from 22 human samples from Accession: GDS3092 Series = GSE5108 (Platform: GPL2895) were investigated. Out of the overall total of 277 404 elements, 1 911 unique elements were identified as differentially expressed and associated with EMS.

### 3.2. DAVID Analysis

Based on the DAVID analysis, the biological characteristics of the 1 911 unique elements were identified, and the genes positively (Table 1) and negatively (Table 2) associated with EMS were determined based on the enrichment score. Positively associated genes have activity that is positively correlated with another gene; these positive correlations indicate functional relationships. Their expression levels are usually calculated based on the Pearson correlation coefficient. On the other hand, directly associated genes and pathways with EMS are presented in Table 3. Directly associated genes have a direct connection between two genes, such as protein-protein interaction or regulatory relationships. They are computationally calculated using PPI databases or transcription factor gene expression databases. The DAVID analysis uses the statistical algorithm EASE (Expression Analysis Systematic Explorer) score, which is essentially a modified Fisher's exact test. The study observed that Symport protein (ES = 34.76), Keratin filament (ES = 32.07), Zinc finger C2H2 (ES = 30.07), Transmembrane transporter (ES = 29.33), and Ribonucleoprotein (ES = 23.88) were positively associated with endometriosis (Table 1). Whereas Krueppel-associated box (ES = 0), Intermediate filament (ES = 0), Homeobox (ES = 0), Leucine-rich repeat (ES = 0), and GTPase (ES = 0) pathways were negatively correlated with endometriosis (Table 2). On the other hand, the genes directly associated with EMS include Cadherin (ES = 14.71), Cyclin (ES = 11.9), Catenin (ES = 8.28), Growth factor receptor binding (ES = 7.79), Mitogen-activated protein kinase (ES = 4.6), serine/threonine kinase (ES = 4.29), EGF Receptor (ES = 1.77), BCL2 (ES = 1.52), EGF (ES = 0.7), and Growth arrest (ES = 0.5) (Table 3). Table 4 presents the diseases most frequently associated with EMS. Cockayne syndrome was mostly associated with EMS (ES = 5.6), followed by Bardet-Biedl syndrome (ES = 3.5), Obesity (ES = 1.9), Diabetes mellitus (ES = 1.6), and Intellectual disability (ES = 1.3). Using the outcomes of text mining as our starting point, we proceeded to create a refined gene set. This was achieved by calculating the probability of observing occurrences beyond what's anticipated for each gene within the subset. In the present study, DAVID analyzed genes were categorized according to the KEGG pathway database, and these pathways were significantly enriched (adjusted P-value < 0.01). The syndrome associated with EMS can be found out using a multidisciplinary approach with the combination of clinical, genetic, and research-based strategies. From multiple medical databases, GWAS (Genome-Wide Association Study) is used to identify genetic factors and potential syndromes associated with endometriosis.

**Table 1.** Positively Associated top 5 Hits Linked with Endometriosis (EMS) Showcasing Correlation Factors ^a^

Identifier	Molecular Function	Count	Fold Change	Benjamini	ES	P-Value	FDR
**Symport**	Sodium ion transmembrane transporter	111	4.5E-75	5.8E0	34.76	2.8E-77	3.9E-75
**Keratin filament **		39	6.9E-26	8.4E-24	32.07	6.9E-26	8.0E-24
**Zinc finger C2H2-type/integrase DNA-binding domain **	Cellular process	289	2.5E0	3.4E-52	30.07	1.6E-55	3.3E-52
**Transmembrane transporter**	Substance transfer	111	4.9E0	3.7E-53	29.33	2.1E-56	3.4E-53
**Ribonucleoprotein**	Ribosomal protein	90	2.2E0	1.2E-12	23.88	6.7E-14	9.1E-13

Abbreviations: FDR, false discovery rate; ES, Enrichment Score.

^a^ The table presents key information including molecular function, gene count, fold change, Benjamini value, enrichment score, P-value, and false discovery rate.

**Table 2.** Negatively Associated top 5 Hits Linked with Endometriosis (EMS) Showcasing Correlation Factors ^a^

Identifier	Function	Count	Fold Change	Benjamini	ES	P-Value	FDR
**Krueppel-associated box**	Transcriptional repression	6	1.3E-1	1.0E0	0	1.0E0	1.0E0
**Intermediate filament**	Cytoskeleton & Nuclear envelope	5	2.7E-1	1.0E0	0	1.0E0	1.0E0
**Homeobox**	DNA binding	9	3.3E-1	1.0E0	0	1.0E0	1.0E0
**Leucine-rich repeat**		17	4.3E-1	1.0E0	0	1.0E0	1.0E0
**GTPase**		5	2.4E-1	1.0E0	0	1.0E0	1.0E0

Abbreviations: FDR, false discovery rate; ES, Enrichment Score.

^a^ The table presents key information including molecular function, gene count, fold change, Benjamini value, enrichment score, P-value, and false discovery rate.

**Table 3.** Top 10 Hits Associated with Endometriosis (EMS) ^a^

Identifier	Count	Fold Change	Benjamini	ES	P-Value	FDR
**Cadherin**	49	3.4E0	4.8E-13	14.71	5.8E-15	4.5E-13
**Cyclin**	31	4.9E0	1.1E-14	11.9	2.2E-16	9.0E-15
**Catenin**	25	6.3E0	4.7E-14	8.28	1.0E-16	4.4E-14
**Growth factor receptor binding (GRB)**	21	6.0E0	1.2E-11	7.79	9.4E-14	1.1E-11
**Mitogen-activated protein kinase**	10	7.3E0	7.7E-6	4.6	1.5E-7	7.2E-6
**serine/threonine kinase**	73	1.1E0	6.7E-1	4.29	1.5E-1	6.1E-1
**EGF Receptor**	43	1.8E0	1.5E-3	1.77	1.2E-4	1.3E-3
**BCL2**	7	4.2E0	6.0E-2	1.52	3.3E-3	5.7E-2
**EGF**	37	1.1E0	1.0E0	0.7	3.9E-1	9.5E-1
**Growth arrest**	4	1.5E0	1.0E0	0.5	5.3E-1	9.3E-1

Abbreviations: FDR, false discovery rate; ES, Enrichment Score.

^a^ These annotations offer insights into the molecular functions associated with genes related to endometriosis.

**Table 4.** Diseases Most Frequently Associated with Endometriosis (EMS) ^a^

Disease	Count	%	Enrichment Score	Benjamani	P-Value	FDR
**Cockayne syndrome**	6	0.2	5.6	3.8E-2	9.3E-4	3.8E-2
**Bardet-Biedl syndrome**	13	0.4	3.5	8.5E-3	7.7E-5	8.5E-3
**Obesity**	18	0.6	1.9	4.0E-1	1.1E-2	4.0E-1
**Diabetes mellitus**	18	0.6	1.6	1.0E0	6.1E-2	1.0E0
**Intellectual disability**	135	4.5	1.3	2.4E-2	4.3E-4	2.4E-2

Abbreviation: FDR, false discovery rate.

^a^ The table provides an overview of diseases frequently associated with EMS, including the count, percentage, enrichment score, Benjamani value, P-value, and false discovery rate.

### 3.3. PPI Network Construction

**Figure 1.**
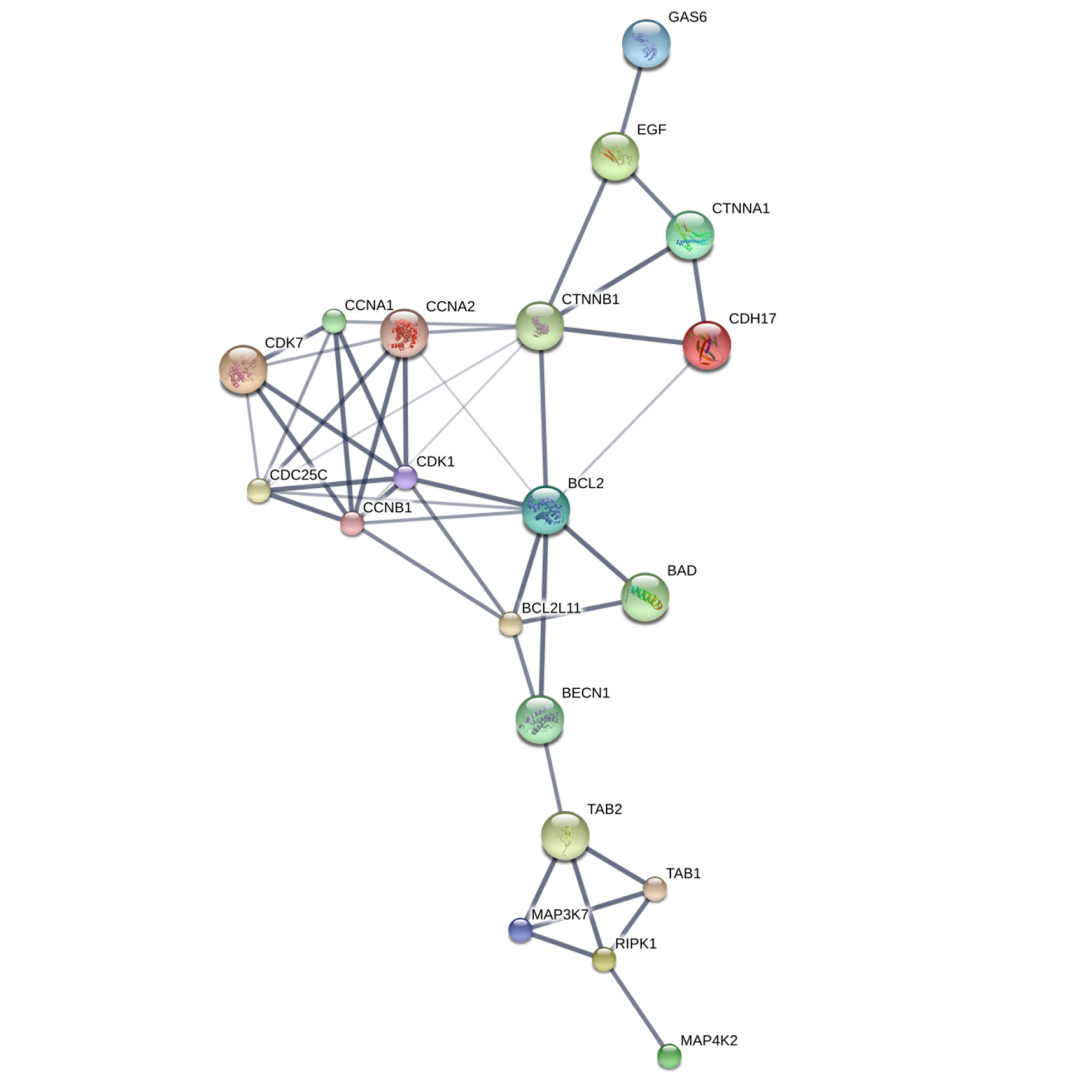
Protein-protein interaction (PPI) interaction network of the HUB Genes from STITCH database.

To gain insights into the interactions among the overlapping DEGs, a PPI network was constructed using the STITCH database. The resulting PPI network was visualized using Cytoscape software version 3.8.2. The degree values of the DEGs were calculated and ranked, identifying the hub genes with higher degree values that are more likely to be associated with EMS. We identified 46 genes exhibiting close interactions with each other, achieving a confidence score of 0.471. However, when applying a cutoff of > 0.713, only 19 genes were retained in the analysis. The hub genes associated with EMS were identified, as depicted in Figure 1. Additionally, the top target proteins were listed in Table 5. These hub genes and enzymes represent potential key players and pathways associated with EMS.

**Table 5.** HUB Genes from STITCH Database Associated with Endometriosis (EMS) with Their Roles ^a^

Identifier	Name	PDB ID	Confidence score	Possible Role
**BAD **	BCL2-associated agonist of cell death	NA	0.962	Promotes cell death.
**BCL2**	B-cell CLL/lymphoma 2	2Xa0 (ChainA)	0.944	Suppresses apoptosis in a variety of cell systems
**BCL2L11**	BCL2-like 11	NA	0.840	Apoptosis facilitator
**BECN1**	Beclin 1, autophagy related;	NA	0.866	Plays a central role in autophagy
**CCNA1**	Cyclin A1	NA	0.997	Involved in the control of the cell cycle at the G1/S
**CCNA2**	Cyclin A2	2x1n	0.898	Essential for the control of the cell cycle at the G1/S
**CCNB1**	Cyclin B1	NA	0.993	Essential for the control of the cell cycle at the G2/M
**CDC25C**	Cell division cycle 25 homolog C (S. pombe)	NA	0.713	Functions as a dosage-dependent inducer
**CDH17**	Cadherin 17	NA	0.949	Calcium-dependent cell adhesion proteins.
**CDK1**	Cyclin-dependent kinase 1	NA	0.999	Plays a key role in the control of the eukaryotic cell cycle by modulating the centrosome cycle
**CDK7**	Cyclin-dependent kinase 7	1ua2	0.957	Involved in cell cycle control and in RNA polymerase II-mediated RNA transcription.
**CTNNA1**	Catenin (cadherin-associated protein), alpha 1, 102kDa	NA	0.934	Associates with the cytoplasmic domain of a variety of cadherins
**CTNNB1**	Catenin (cadherin-associated protein), beta 1, 88kDa	NA	0.987	Key downstream component of the canonical Wnt signaling pathway.
**EGF**	Epidermal growth factor	1nql	0.900	EGF stimulates the growth of various epidermal and epithelial tissues
**GAS6**	Growth arrest-specific 6	1h30 (Chain A)	0.900	Implicated in cell growth and survival, cell adhesion and cell migration.
**MAP3K7**	Mitogen-activated protein kinase kinase kinase 7	NA	0.996	
**RIPK1**	Receptor (TNFRSF)-interacting serine-threonine kinase 1	NA	0.995	Transduces inflammatory and cell-death signals (programmed necrosis) f
**TAB1**	TGF-beta activated kinase 1	NA	0.969	Play an important role in mammalian embryogenesis
**TAB2**	TGF-beta activated kinase 2	2wwz (Chain C)	0.995	Promotes MAP3K7 activation in the IL1 signaling pathway.

^a^ Key molecular players associated with EMS, providing information on PDB ID (if available), confidence score, and possible roles in cellular processes are presented.

### 3.4. Molecular Docking Analysis

**Figure 2.**
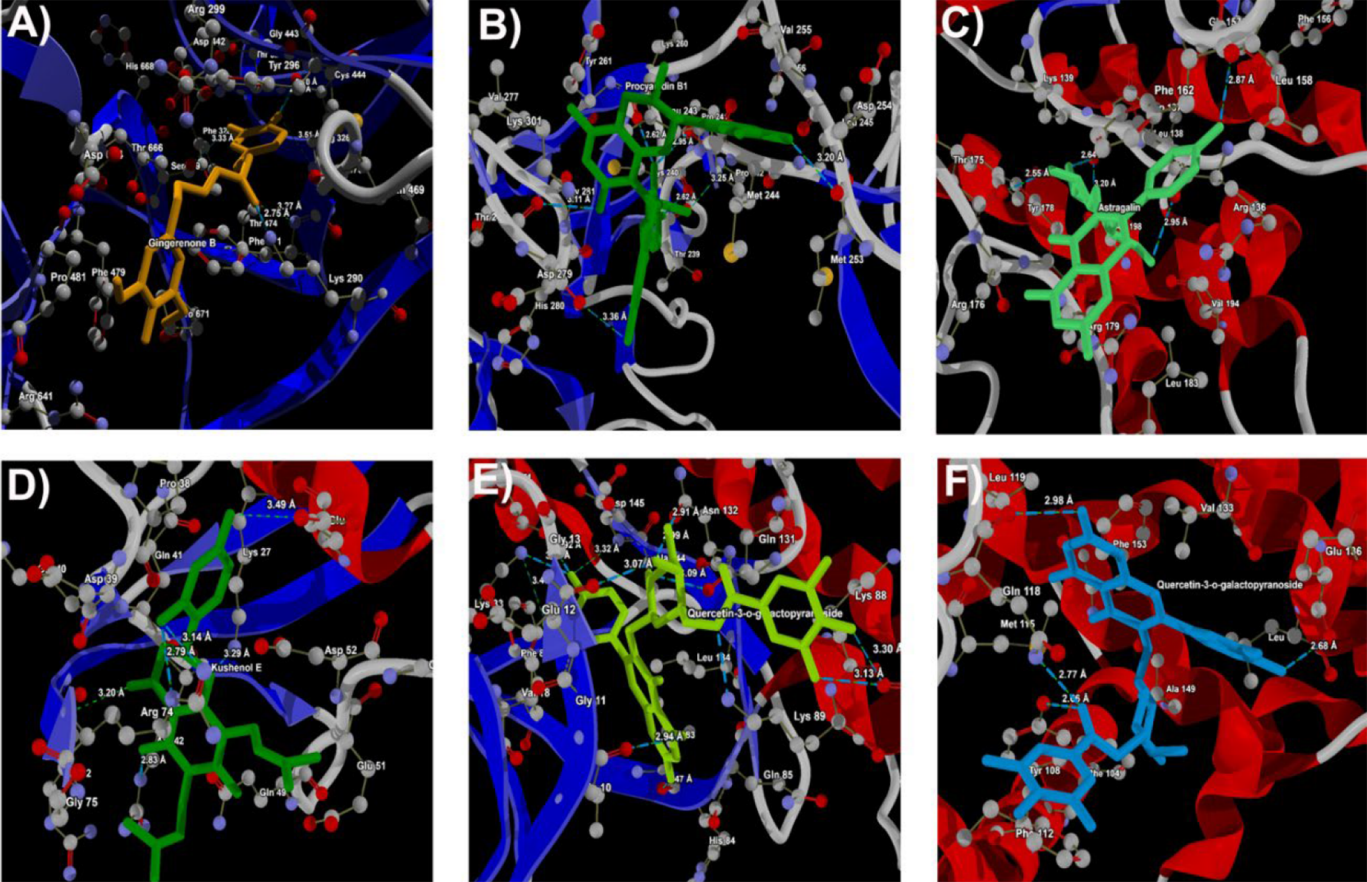
Docking of A, gingerenone B at the active site of 1H30; B, procyanidin B1 at the active site of 1NQL; C, astragalin at the active site of 1UA2; D, kushenol E at the active site of 2WWZ, and Quercetin-3-o-galactopyranoside at the active site of; E, 2X1N and; F, 2XA0 respectively.

**Figure 3.**
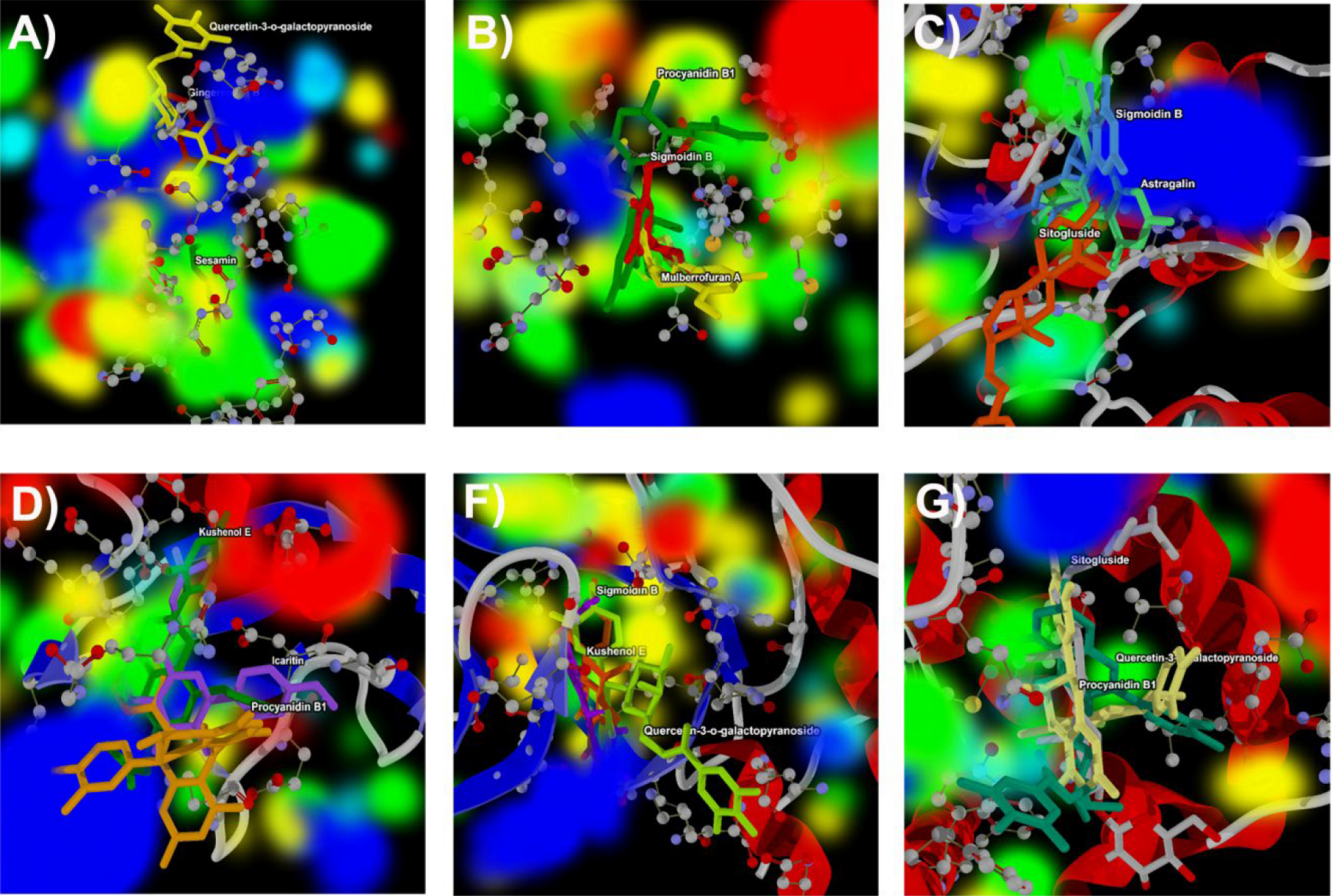
Energy map analysis of the top three docking hits against; A, gingerenone B, sesamin, and Quercetin-3-o-galactopyranoside at the active site of 1H30; B, Procyanidin B1, Mulberrofuran A, and Sigmoidin B at the active site of 1NQL; C, astragalin, Sitogluside, and Sigmoidin B at the active site of 1UA2; D, kushenol E, Procyanidin B1, and Icaritin at the active site of 2WWZ; E, quercetin-3-o-galactopyranoside, Kushenol E, and Sigmoidin B at the active site of 2X1N; and F, quercetin-3-o-galactopyranoside, Procyanidin B1, and Sitogluside at the active site of 2XA0 respectively. Green color indicates the region that might contribute to steric interaction, turquoise color indicates hydrogen acceptor favorable regions, yellow indicates hydrogen donor favorable regions, and electrostatic potential regions with red and blue color.

In this investigation, we present the docking scores and key interaction properties of the top 10 ligands docked at the active site of the target proteins, namely PDB IDs: 1H30 (Table 6), 1NQL (Table 6), 1UA2 (Table 7), 2WWZ (Table 7), 2X1N (Table 8), and 2XA0 (Table 8). The molecular interaction analysis of the top docking hits against the target proteins associated with endometriosis is detailed in Figure 2. The molecular interactions map of Gingerenone B at the active site residues of GAS6 (PDB ID: 1H30) is demonstrated in Figure 2A, while Figure 2B depicts the interactions of Procyanidin with the active site residues of EGF (PDB ID: 1NQL). Astragalin and Kushenol E also exhibit strong molecular interactions at the active site of CDK7 (PDB ID: 1UA2) and TAB2 (PDB ID: 2WWZ) respectively, as shown in Figure 2C and D. Figure 2E and F represent the molecular interaction map of Quercetin-3-o-galactopyranoside at the active site of CCNA2 (PDB ID: 2X1N) and BCL2 (PDB ID: 2XA0) respectively. Figure 3A - F illustrate the energy map of endometrial-associated proteins, indicating contributions to favorable steric interactions (depicted in green), hydrogen acceptor preferences (shown in turquoise), hydrogen donor preferences (represented in yellow), and the electrostatic potential of PDB IDs: 1H30, 1NQL, 1UA2, 2WWZ, 2X1N, and 2XA0. Each map corresponds to the top three docking hits for the ligands associated with each target protein.

**Table 6.** Docking Results of top 10 Docking Hits of 1H30 and 1NQL ^a^

PDB and Ligand	MolDock Score	Rerank Score	Interaction	HBond	Total
**1H30**					
Gingerenone B	-127.09	-102.84	-163.32	-4.51	-397.77
Sesamin	-133.83	-79.18	-136.07	-8.77	-357.85
Quercetin-3-o-galactopyranoside	-87.98	-88.63	-137.90	-11.97	-326.49
Moracin D	-114.52	-76.18	-124.08	-8.51	-323.28
Alexandrin	-92.31	-71.10	-131.31	-5.60	-300.32
Mulberrofuran A	-100.29	-77.12	-114.25	-1.96	-293.62
Astragalin	-81.96	-76.46	-114.49	-12.40	-285.31
Quercetin	-89.30	-70.07	-114.53	-7.12	-281.01
Campesterol	-93.43	-71.60	-115.14	0.00	-280.17
Icaritin	-81.89	-74.72	-109.06	-3.29	-268.96
**1NQL**					
Procyanidin B1	-136.94	-115.57	-168.94	-9.73	-431.17
Mulberrofuran A	-120.75	-94.89	-132.47	-2.50	-350.61
Sigmoidin B	-105.95	-92.33	-130.41	-4.97	-333.66
Kushenol E	-97.78	-90.73	-127.27	-5.00	-320.78
Astragalin	-100.41	-89.62	-123.37	-10.00	-323.40
Beta-Sitosterol	-110.92	-88.04	-120.61	-2.35	-321.91
Kadsurin A	-100.92	-86.59	-121.54	-0.75	-309.80
Quercetin-3-o-galactopyranoside	-78.87	-81.84	-134.43	-11.80	-306.95
Aureusidin	-93.71	-80.87	-104.05	-6.05	-284.68
Arctigenin	-96.82	-80.30	-124.64	-2.50	-304.27

^a^ Scores such as MolDock Score, Rerank Score, Interaction, H-Bond, and Total score provide insights into the binding affinities and interaction profiles of ligands.

**Table 7.** Docking Results of top 10 Docking Hits of 1UA2 and 2WWZ ^a^

PDB and Ligand	MolDock Score	Rerank Score	Interaction	HBond	Total
**1UA2**					
Astragalin	-122.10	-112.04	-155.65	-10.75	-400.54
Sitogluside	-110.44	-90.10	-150.16	-10.84	-361.55
Sigmoidin B	-112.50	-98.56	-136.34	-5.04	-352.44
Moracin D	-117.45	-97.35	-127.42	-6.65	-348.86
Galgravin	-112.25	-88.77	-134.57	-2.49	-338.07
Mulberrofuran A	-111.09	-88.39	-129.19	-5.08	-333.75
alpha1-Sitosterol	-111.97	-91.30	-127.04	0.00	-330.31
Kadsurin A	-110.84	-92.65	-125.65	-0.47	-329.61
Rhamnazin	-101.90	-86.78	-133.74	-6.21	-328.62
Gingerenone B	-102.55	-88.69	-130.95	-4.98	-327.18
**2WWZ**					
Kushenol E	-118.92	-99.03	-144.71	-7.78	-370.44
Procyanidin B1	-117.52	-89.11	-139.46	-13.84	-359.94
Icaritin	-113.54	-98.83	-140.02	-5.41	-357.80
Episyringaresinol	-114.67	-94.31	-137.47	-4.69	-351.15
Quercetin-3-o-galactopyranoside	-89.49	-91.99	-151.81	-11.44	-344.73
Mulberrofuran A	-111.97	-81.77	-122.68	-5.86	-322.28
Scopolin	-101.84	-77.98	-128.43	-13.70	-321.96
Phaseol	-110.79	-86.98	-120.61	-3.51	-321.89
Sigmoidin B	-99.27	-86.14	-123.78	-7.52	-316.71
Glycyrol	-102.38	-82.22	-117.57	-6.69	-308.86

^a^ Scores such as MolDock Score, Rerank Score, Interaction, H-Bond, and Total score provide insights into the binding affinities and interaction profiles of ligands.

**Table 8.** Docking results of top 10 docking hits of 2X1N and 2XA0 ^a^

PDB and Ligand	MolDock Score	Rerank Score	Interaction	HBond	Total
**2X1N**					
Quercetin-3-o-galactopyranoside	-125.30	-125.65	-187.46	-20.17	-458.58
Kushenol E	-127.11	-113.63	-152.74	-9.17	-402.64
Sigmoidin B	-126.48	-111.16	-150.33	-13.94	-401.91
Mulberrofuran A	-137.57	-109.82	-148.86	-2.02	-398.27
Arctigenin	-128.78	-105.23	-142.28	-4.34	-380.63
Licoflavonol	-117.25	-109.08	-145.93	-7.36	-379.62
Sitogluside	-118.77	-98.61	-157.28	-4.15	-378.81
Glycyrol	-120.29	-105.09	-138.97	-4.94	-369.29
Astragalin	-106.89	-103.74	-146.31	-9.74	-366.68
Phaseol	-124.67	-104.51	-134.33	-2.50	-366.01
**2XA0**					
Quercetin-3-o-galactopyranoside	-90.58	-86.87	-142.10	-6.29	-325.84
Procyanidin B1	-113.08	-76.83	-125.56	-8.35	-323.83
Sitogluside	-99.00	-85.65	-122.43	0.00	-307.08
Kushenol E	-95.33	-82.77	-118.94	-2.50	-299.54
Astragalin	-94.86	-80.67	-109.77	-10.00	-295.30
Sesamin	-104.48	-77.83	-106.39	-1.43	-290.13
Moracin D	-97.42	-81.05	-107.28	-3.72	-289.46
Glycyrol	-95.99	-80.34	-108.72	-3.82	-288.87
Mulberrofuran A	-97.32	-79.13	-110.80	0.00	-287.25
Gingerenone B	-88.69	-74.76	-120.70	-2.42	-286.56

^a^ Scores such as MolDock Score, Rerank Score, Interaction, H-Bond, and Total score provide insights into the binding affinities and interaction profiles of ligands.

In Table 6, Gingerenone B and Sesamin exhibit the most favorable score against 1H30. These compounds show strong interactions, as indicated by their Rerank Scores, with Sesamin forming the highest number of hydrogen bonds. Procyanidin B1 emerges as the top compound against 1NQL, displaying the most favorable score (Table 6), with Mulberrofuran A and Sigmoidin B also showing notable docking scores and significant interaction and hydrogen bonding. Astragalin and Sitogluside are prominent ligands against 1UA2, exhibiting the most favorable scores and substantial interactions with hydrogen bonding, suggesting their potential in binding to the target (Table 7). Kushenol E and Procyanidin B1 also demonstrate strong docking affinities against 2WWZ, with notable interactions and hydrogen bonding, while Quercetin-3-o-galactopyranoside and Icaritin show promising docking results (Table 7). In Table 8, Quercetin-3-o-galactopyranoside and Kushenol E are the top-performing ligands for 2X1N, with Quercetin-3-o-galactopyranoside exhibiting the most favorable Score and forming a high number of hydrogen bonds, contributing to their favorable docking results. Similarly, in Table 8, Quercetin-3-o-galactopyranoside and Procyanidin B1 emerge as top ligands against 2XA0, displaying favorable Scores, considerable interactions, and hydrogen bonding, along with notable docking affinities of Sitogluside and Kushenol E.

## 4. Discussions

In this study, we meticulously curated a set of 1 911 elements extracted from 22 human samples present in GDS3092, Series = GSE5108 (Platform: GPL2895). Through analysis, it was uncovered that among these 1 911 elements, a few distinct ones displayed specific differential expression patterns associated with endometriosis (EMS). While the precise origins of endometriosis remain elusive, numerous pivotal genes and pathways have emerged as contributors to its advancement. In our current investigation, pivotal genes encompassing BCL2, CCNA2, CDK7, EGF, GAS6, MAP3K7, and TAB2 were identified as central hub genes (14). This study highlights genes intricately linked with EMS, comprising Cadherin, Cyclin, Catenin, Growth Factor Receptor Binding, Mitogen-Activated Protein Kinase, Serine/Threonine Kinase, EGF Receptor, BCL2, EGF, and Growth Arrest. Wang et al. utilized advanced bioinformatics methodologies to unveil potential pathways and pivotal genes underpinning endometriosis progression (15). Their analyses, employing GO and KEGG, revealed that the identified similar genes were notably enriched in protein interactions, cellular barrier reinforcement, and cellular life dynamics (15). In a separate study, 22 endometriosis-related immune genes emerged from the overlap of 1 116 DEGs, featuring nine immune-related hub genes (BST2, CCL13, CD86, CSF1, FAM3C, GREM1, ISG20, PSMB8, S100A11) and nine ARG hub genes (GSK3A, HTR2B, RAB3GAP1, ARFIP2, BNIP3, CSF1, MAOA, PPP1R13L, SH3GLB2) (16). The heightened expression of these hub genes is intricately linked to diverse processes, including DNA methylation, protein thiol-disulfide exchange, chromatin silencing, myosin thick filament formation, and systemic development (17). Moreover, these hub genes are intricately linked to various supplementary signaling pathways, aiming to unveil densely interconnected regions within the network structure. This endeavor facilitates the identification of cohesive molecular complexes (16). The ectopic EMS revealed twelve hub genes, while the eutopic EMS identified sixteen, both of which were subsequently validated in independent datasets (10). In a separate study, employing a predefined threshold, five pivotal genes—TP53 (tumor protein p53), VEGFA (vascular endothelial growth factor A), AKT1 (v-akt murine thymoma viral oncogene homolog 1), MMP9 (matrix metallopeptidase 9), and IL6 (interleukin 6)—were unveiled as associated with EMS (18). Through meticulous curation involving labor-intensive efforts, the study identified a total of 1 911 genes directly linked to endometriosis. This curated gene set underwent rigorous statistical refinement, resulting in a highly reliable compilation of endometriosis-related genes (19). In our study, only seven hub genes with a 3D structure were identified, namely BCL2, CCNA2, CDK7, EGF, GAS6, MAP3K7, and TAB2 (14). The study also reveals a significant disparity in the number of DEGs, prompting a reassessment of the histological origin of the ectopic endometrium. Additionally, the seven hub genes with PDB structures play an important role in the protein-protein interaction network (20).

Researchers continue to explore these molecular mechanisms to achieve deeper information about the disease and the progression of potential targeted treatments. Tables in the results section provide the top hits associated with endometriosis positively as well as negatively. In the positively associated hits, the fold changes for each category indicate substantial impacts on gene expression. Enrichment scores are notably high, indicating strong associations with specified molecular functions. On the other hand, the fold changes are relatively low, suggesting subtle changes in gene expression. The Benjamini values indicate that none of the associations are statistically significant, and O enrichment scores in all the hits further suggest a lack of significant enrichment in specified functions. The P-values and FDR values are consistently high, reinforcing the lack of statistical significance.

These findings hold potential for advancing our understanding of endometriosis. A total of 1 911 differentially expressed genes (CDGs) emerged in the study's three pairwise comparisons. Integrative bioinformatics studies identified DEGs as promising candidates for diagnostic biomarkers and therapeutic targets in endometriosis (21). The genes associated with EMS span pathways such as Symport, Keratin filament, Zinc finger C2H2, Transmembrane transporter, and Ribonucleoprotein (22). Genetic evaluation of DEGs was performed using the DAVID database (12), a unifying framework synthesizing data from various functional annotations from diverse sources. Differential gene expression analysis was conducted, applying criteria of a 5% adjusted P-value and a 2.0-fold change threshold. Pathways were then determined through functional enrichment using the Molecular Signatures Database, considering a P-value < 5% and an FDR q-value of ≤ 25%. Genes that played a more recurrent role in pathways were identified utilizing leading-edge analysis (22). Contrarily, gene chip technology offers an efficient, high-capacity method for simultaneous tissue-wide or organism-wide gene expression assessment (23). This capability positions it as an effective tool for promptly detecting disease-linked genes and identifying potential biomarkers (24). Comprehensive KEGG and GO analysis revealed enriched cellular communication pathways closely tied to inflammatory processes, complement initiation, cell connection, and the external medium within endometriosis-linked cell groups. Drawing insights, we propose that endometriosis progression involves TLR4/NF-κB, Wnt/frizzled signaling pathways, and estrogen receptors—promising targets for both therapeutic interventions and diagnostic approaches (25). In the vascular arrangement and endometria of the uterus, estrogen acceptors closely collaborate with NF-κB, governing enzyme function in prostaglandin synthesis, including cyclooxygenase enzyme 2 and Prostaglandin I2 synthase (26). In this study, docking results showed potential ligand-protein interactions associated with EMS. Noteworthy ligands like Quercetin-3-o-galactopyranoside, Kushenol E, Procyanidin B1, Mulberrofuran A, and Astragalin consistently exhibit strong binding across multiple simulations, indicated by low MolDock Scores and hydrogen bonding. Sesamin and Gingerenone B show promise against 1H30. Procyanidin B1 exhibits the highest negative scores for potent binding to 1NQL, while Mulberrofuran A and Sigmoidin B display notable negative scores. Quercetin-3-o-galactopyranoside parallels Astragalin, and Astragalin has the lowest MolDock Score against 1UA2. Galgravin and Mulberrofuran A exhibit strong binding, and Kushenol E has the lowest MolDock Score against 2WWZ, with consistent post-refinement results. Quercetin-3-o-galactopyranoside shows robust binding with unique profiles against 2WWZ, supported by lower MolDock Score, high Interaction score, and numerous hydrogen bonds, offering crucial insights for further investigation into EMS ligand-protein interactions. This discovery is crucial in drug design, as hydrogen bonds enhance molecular interaction specificity and strength. The identified ligands, especially those consistently effective, emerge as promising candidates for further exploration in developing EMS therapeutics. Consequently, a thorough investigation is imperative to explore the theory regarding the cellular origin of endometriosis.

Boje et al.'s recent cohort study revealed three primary findings: Increased pregnancy losses with higher euploid probability among women with endometriosis, reduced pregnancy achievement rates in affected women, and a clear correlation between endometriosis and pregnancy loss, intensifying with an escalating number of losses (27). The outcomes underscored a notable convergence of genes demonstrating heightened activity within pathways like the cyclin A1 pathway, cyclin-dependent kinase, Epidermal growth factor, MAP TAB signaling pathway, and other pathways commonly linked with solid cancers (28). Liu et al. used STRING and Cytoscape to construct a PPI network, identifying 160 DEGs, with 51 upregulated. Within this network, 100 DEGs were found, and three genes (BIRC5, CENPF, HJURP) overlapping with DEM targets were associated with worse overall survival in endometrial cancer (29). In contrast, Zheng et al. found 687 DEGs in endometriosis involving cell adhesion, MAPK, PI3K-Akt, cytokine receptors, and EMT pathways. Pale turquoise module hub genes (e.g., FOSB, JUNB) are linked to TNF, MAPK, foxO, oxytocin, and p53 pathways, suggesting roles in immune response, stem cell self-renewal, and epithelial-mesenchymal transformation (30). Another study revealed upregulated genes like EGF and IL-1β in endometriosis, associated with focal adhesion and calcium signaling, implicating them in endometriosis pathogenesis (31). However, based on the microarray data set, Cockayne syndrome, Bardet-Biedl syndrome, Obesity, Diabetes mellitus, and Intellectual disability were mostly associated with endometriosis. With time, they could proliferate and contribute to the development of endometriosis. If an individual suspects that they have endometriosis or any related symptoms, it's recommended to consult a medical professional for proper diagnosis and management.

### 4.1. Conclusions

In conclusion, this comprehensive analysis of microarray data and subsequent DAVID analysis provided valuable insights into the molecular landscape of EMS. The identification of 1 911 DEGs provides a foundation for understanding the molecular basis of this intricate disorder. The utilization of the DAVID elucidates biological pathways positively and negatively associated with endometriosis, shedding light on the intricate genetic networks involved. The PPI network analysis reveals hub genes, including BCL2, CCNA2, CDK7, EGF, GAS6, MAP3K7, and TAB2, which emerge as pivotal players in endometriosis progression. These findings align with existing literature, emphasizing the importance of these genes in the context of endometriosis. The molecular docking simulations further contribute by identifying potential ligands, such as Quercetin-3-o-galactopyranoside and Kushenol E, displaying favorable interactions with target proteins associated with endometriosis. The consistent performance of these ligands across multiple targets suggests their broad-spectrum effectiveness, warranting further exploration in therapeutic interventions. These findings contribute to our understanding of the molecular mechanisms underlying EMS and offer promising avenues for further research and therapeutic development in addressing this complex condition.

## Data Availability

The dataset presented in the study is available on request from the corresponding author during submission or after publication.
